# Investigating the Bond Strength of FRP Laminates with Concrete Using LIGHT GBM and SHAPASH Analysis

**DOI:** 10.3390/polym14214717

**Published:** 2022-11-03

**Authors:** Muhammad Nasir Amin, Babatunde Abiodun Salami, Muhammad Zahid, Mudassir Iqbal, Kaffayatullah Khan, Abdullah Mohammad Abu-Arab, Anas Abdulalim Alabdullah, Fazal E. Jalal

**Affiliations:** 1 Department of Civil and Environmental Engineering, College of Engineering, King Faisal University, Al-Ahsa 31982, Saudi Arabia; 2 Interdisciplinary Research Center for Construction and Building Materials, Research Institute, King Fahd University of Petroleum and Minerals, Dhahran 31261, Saudi Arabia; 3 Department of Civil, Geological and Mining Engineering, École Polytechnique de Montréal, Montreal, QC H3C 3A7, Canada; 4 Department of Civil Engineering, University of Engineering and Technology, Peshawar 25120, Pakistan; 5 Department of Civil Engineering, Shanghai Jiao Tong University, Shanghai 200240, China

**Keywords:** FRP, interfacial bond strength, statistical analyses, LIGHT GBM, XGBoost, ensemble models, SHAPASH analysis

## Abstract

The corrosion of steel reinforcement necessitates regular maintenance and repair of a variety of reinforced concrete structures. Retrofitting of beams, joints, columns, and slabs frequently involves the use of fiber-reinforced polymer (FRP) laminates. In order to develop simple prediction models for calculating the interfacial bond strength (IBS) of FRP laminates on a concrete prism containing grooves, this research evaluated the nonlinear capabilities of three ensemble methods—namely, random forest (RF) regression, extreme gradient boosting (XGBoost), and Light Gradient Boosting Machine (LIGHT GBM) models—based on machine learning (ML). In the present study, the IBS was the desired variable, while the model comprised five input parameters: elastic modulus x thickness of FRP (*E_f_T_f_*), width of FRP plate (*b_f_*), concrete compressive strength (*f_c_*′), width of groove (*b_g_*), and depth of groove (*h_g_*). The optimal parameters for each ensemble model were selected based on trial-and-error methods. The aforementioned models were trained on 70% of the entire dataset, while the remaining data (i.e., 30%) were used for the validation of the developed models. The evaluation was conducted on the basis of reliable accuracy indices. The minimum value of correlation of determination (R^2^ = 0.82) was observed for the testing data of the RF regression model. In contrast, the highest (R^2^ = 0.942) was obtained for LIGHT GBM for the training data. Overall, the three models showed robust performance in terms of correlation and error evaluation; however, the trend of accuracy was obtained as follows: LIGHT GBM > XGBoost > RF regression. Owing to the superior performance of LIGHT GBM, it may be considered a reliable ML prediction technique for computing the bond strength of FRP laminates and concrete prisms. The performance of the models was further supplemented by comparing the slopes of regression lines between the observed and predicted values, along with error analysis (i.e., mean absolute error (MAE), and root-mean-square error (RMSE)), predicted-to-experimental ratio, and Taylor diagrams. Moreover, the SHAPASH analysis revealed that the elastic modulus x thickness of FRP and width of FRP plate are the factors most responsible for IBS in FRP.

## 1. Introduction

Repairing and reinforcing structures has traditionally been a dynamic and complex aspect of building work. The use of fiber-reinforced polymer (FRP) bars, sheets, and strips to enhance RC or even steel structural components is one of the prevalent approaches for these kinds of repairs. Because of the corrosion of traditional steel reinforcement that leads to deterioration, RC structures require periodic maintenance and repair [1,2,3]. As a result, to meet the strength requirements and revised code designs, old structures are being strengthened as a newly emerging construction activity [4]. FRP laminates (FRP sheets) are multilayer sheets with directional resistance that are glued to concrete or steel surfaces using specific FRP epoxy resin systems. Because of their better performance, they are extensively used for structural retrofitting and repair of insufficiently reinforced concrete structures [5,6,7]. Owing to their characteristics such as light weight, great resistance to corrosion and chemicals, higher creep/fatigue resistance, greater tensile strength, high stiffness, and ease of application, FRP is one of the most successful approaches to reinforcing concrete buildings [2,8,9,10]. Although such materials have been effectively employed for reinforcement, as mentioned above, the problem of bonding as well as delamination in all types of systems continues to be worrisome, as this method is dependent on the quality of stress transmission at the concrete surface or the depth of concrete cover. FRP-strengthened structures are susceptible to a variety of failure types, such as FRP rupture, concrete crushing, and shear cracks, among others. The ultimate rupture strength of FRP sheets can be improved by surface treatment prior to bonding [1]. Premature debonding can also occur prior to approaching the final capacity, with debonding of the FRP laminate spreading from one end to the center being the most commonly recorded failure [11,12]. Debonding may intensify at elevated temperatures, resulting in a decrease in the structural capacity of the RC structures. Furthermore, the interfacial bond failures are influenced by the bond’s quality owing to manufacturing [2,13,14]. 

Recent laboratory studies indicate that the potential for brittle debonding failures is alarming in FRP laminates, depending on the composite action in the FRP as well as the concrete prism [15,16]. To improve the bonding between the FRP and concrete, a variety of approaches, such as epoxy interlocking at the surface mounting, can be utilized. Hence, the provision of a reasonable interface between the concrete and the FRP laminate is essential to impart flexure strength to beams.

To attain improved lamination, it is pertinent to mention that the damaged surface layer of the concrete is removed while preparing the surface and exposing the coarse aggregates [17,18,19,20]. Surface treatment leads to consistency at the interface of the FRP sheet and the concrete surface, causing delayed debonding and, thus, resulting in an increased ultimate rupture strength. Sandblasting is performed for the removal of the dust on the uncovered surface of the concrete, using special brushes followed by washing with solvents and drying prior to the installation of FRP sheets [2]. 

**Figure 1:**
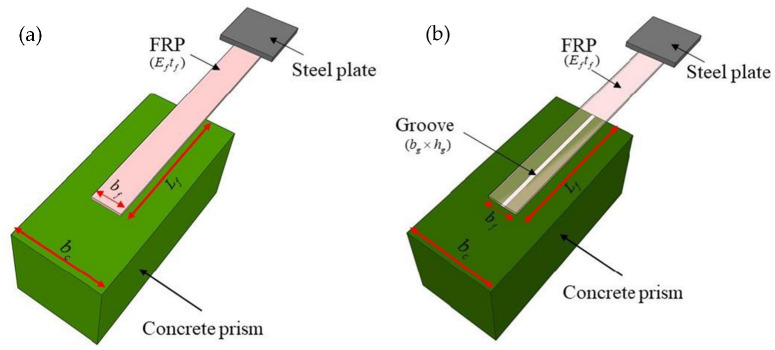
Schematic diagram of a single-lap shear test to compute the interfacial bond strength such that: (**a**) FRP is externally bonded on concrete without groove, and (**b**) FRP is externally attached to the concrete with groove [2].

Single-lap shear tests (SSTs) for FRP laminates that are externally attached to the concrete prism as well as externally attached to its grooves are depicted in Figure 1. The force is exerted unidirectionally on the steel plate at the FRP’s tip such that the structure is fractured. Direct application of FRP on concrete or near-surface mounting technologies, which consist of FRP rebar and laminates inserted in the grooves and subsequently filled with highly adhesive materials, are used for the reinforcement [21,22,23]. Another approach uses FRP laminates that are externally glued to the concrete’s surface grooves. The surface area, accessibility of the material, expense, reliability, and the use of accompanying equipment all contribute to the selection of a particular technique. The relatively high prices, environmental contamination, and facility operating processes to assess the final capacity of specimens prior to their practical application are only a few of the challenges. Because of their dependability and simplicity, a few conventional experimental techniques, such as the SST, have been employed as a basic approach to measure the interfacial bond strength (IBS). Previous research has proposed empirical or semi-empirical formulations for estimating the IBS based on SST experimental data. The suggested models’ empirical relations match the experimental data well; nevertheless, these models have not yet been verified using additional unseen data. Furthermore, in developing these empirical relationships, fundamental simplification assumptions have been made [2,24,25,26,27].

In a variety of engineering challenges, machine learning (ML) or artificial intelligence (AI) is commonly utilized to discover the best solution to regression and classification problems. These ML models are not only trained with a large series of experimental findings, but they are also verified using unseen data. Furthermore, they have seen a wide range of applications in composite constructions, particularly over the past couple of years. This study takes into consideration three AI methods, i.e., Light Gradient Boost Machine (LIGHT GBM), extreme gradient boosting (XGBoost), and random forest (RF) regression. Liang et al. [28] predicted the creep performance of concrete by utilizing LIGHT GBM, XGBoost, and RF algorithms. Note that a data-driven model performs prediction on the basis of specific input data to (a) develop understanding of model decisions, (b) determine the complex hidden nonlinear relationships, and (c) evaluate the implications of a model’s analysis and evaluation. Mangalathu et al. [29] used a wide database in order to analyze the feature importance for the failure mode of RC structural elements, i.e., columns and shear walls. An RF model was formulated for the training set such that it possessed an accuracy of 84% and 86% for the unseen data of the two types of RC elements, respectively. Milad et al. [30] collected an experimental dataset comprising 729 experimental values to predict the FRP strain such that the governing input factors were material geometry, strength characteristics, strain characteristics, FRP characteristics, and confinement characteristics. They deployed XGBoost, RF, and multivariate adaptive regression splines (MARS) algorithms and found that the latter model exhibited the highest prediction accuracy. Xu et al. [31] concluded that the XGBoost model yielded the best model performance and outperformed the empirical models, as well as the RF, decision tree (DT), and artificial neural network (ANN) algorithms. Kim et al. [32] presented four ensemble ML approaches (i.e., CatBoost, histogram gradient boosting, XGBoost, and RF algorithms) for the estimation of FRP–concrete interfacial bond strength (IBS) by considering an extensive dataset with the results of 855 SSTs on the FRP–concrete IBS. They found that the CatBoost algorithm outperformed all of the other ensemble techniques (R^2^ = 0.96, and other performance metrics were also lower). Su et al. [24] used multilinear regression (MLR), support-vector machine (SVM), and ANN models to estimate the IBS of FRP laminates to the concrete prism, and they achieved an R2 of 0.85 for the overall dataset. In yet another study on the estimation of seismic performance of RC walls, Zhang et al. [33] revealed that the XGBoost and gradient boost (GB) algorithms were efficacious, achieving an accuracy of almost 97%, whereas, the GB and RF regression methods performed best in forecasting the lateral strength and ultimate drift ratio of the RC walls. Liu [34] conducted a study by utilizing XGBoost, RF, and support-vector regression (SVR) algorithms for the strength prediction of high-performance concrete. With the help of data preprocessing as well as parameter optimization, these three techniques yielded a better prediction state (R^2^ > 0.9 for all cases) and good model fitting effect, where XGBoost possessed the highest prediction accuracy. While predicting the creep performance of concrete, Liang et al. [28] modelled the creep data in the Northwestern University (NU) database using LIGHT GBM, XGBoost, and RF techniques. After that, SHapley Additive exPlanations (SHAP) were computed for interpreting the predicted values on the basis of cooperative game theory [35,36]. In contrast, the LIGHT GBM approach was found to attain higher accuracy with a substantially shorter calculation duration. Moreover, this game-theory-based framework (i.e., SHAP) has been efficacious in explaining various supervised learning models [37].

To summarize, for the sake of improving the expense of civil engineering projects, AI models based on known experimental findings are required to estimate the IBS of FRP plates on a concrete prism. Due to highly nonlinear correlations between bond strength and a multitude of contributing parameters, typical prediction models for FRP–concrete coupling need further investigation [38]. The authors of the present study are of the viewpoint that the previously formulated models can be further improved in terms of accuracy. In addition, the parametric analysis is a desideratum for investigating the impact of input variables on the IBS, because it finally makes the decision as to which type of strengthening method is most efficacious and economical. Therefore, the present study investigated the ability of the LIGHT GBM, XGBoost, RF regression models in predicting the interfacial bond strength (IBS) of FRP laminates externally bonded to the grooves of a concrete prism by utilizing 136 experimental SST results (anchorage made on one end of FRP to the concrete prism, as shown in Figure 1b). Tested samples with FRP plates parallel to the groove direction were used in the analysis. 

## 2. Methodology

### 2.1. Overview of LIGHT GBM

LIGHT GBM is a Microsoft open-source gradient boosting machine learning framework that employs a decision tree as a training method [39]. LIGHT GBM reportedly surpasses existing gradient boosting techniques—including gradient boosting decision tree (GBDT) and extreme gradient boosting (XGBoost)—in terms of learning and training speed, as well as prediction accuracy, due to the fact that it employs two novel techniques: exclusive feature bundling (EFB), which is designed to manage multiple characteristics of data while avoiding overfitting issues; and gradient-based one-side sampling (GOSS), which is used for managing huge datasets.

Consider an input data including n instances $s=\left\{\left(x_{1},y_{1}\right),\left(x_{2},y_{2}\right)\ldots ,\left(x_{n},y_{n}\right)\right\},$ where $\left\{x_{1},x_{2}\ldots ,\;x_{n}\right\}\;$are independent variables and $\left\{y_{1},y_{2}\ldots ,\;y_{n}\right\}$ are dependent variables. The dependent variable is the ultimate capacity $\left(p\right)$, and the independent variables are concrete compressive strength $\left(f\left(x\right)\right)$, width of groove $b_{g}$, depth of groove $h_{g}$, width of FRP plate $b_{f}$, and elastic modulus of FRP $E_{f}T_{f}$. The estimated values of GBDT $f\left(x\right)\;$are the summation of the outcomes of a set of decision tree models $h_{t}\left(x\right)$:

(1) $$f\left(x\right)=\overset{T}{\underset{t=1}{\sum }}h_{t}\left(x\right)$$

where T represents the number of trees. Finding an approximation function f that aims to minimize the loss function $L\left(y,f\left(x\right)\right)$ is the main focus of fitting a GBDT method, as shown in Equation (2):

(2) $$\hat{f}=argminE_{y,S}L\left(y,f\left(x\right)\right)\;\;$$

In particular, with regard to adopting GOSS for sampling, LIGHT GBM leverages the EFB to accelerate the training procedure without compromising precision. Several applications include attributes that are mutually incompatible, such as high-dimensional and limited inputs. Such attributes can be aggregated into a single attribute bundle by EFB. To use an attribute scanning method, the statistics of such attribute bundles and specific attributes can be compiled. In brief, LIGHT GBM is a new machine learning (ML) method that employs GOSS for internal node splits based on variability gain and EFB to reduce the dimensions of the input attributes. As a decision-tree-based approach, LIGHT GBM has the significant benefit of being sensitive to multicollinearity [39]. Therefore, the incorporation of correlated predictors or independent attributes, which is highly prevalent in concrete data, is not problematic in the LIGHT GBM model.

### 2.2. Model Development

The optimization of hyperparameters is a crucial step in training machine learning (ML) techniques, as it can improve the generalization and prediction robustness of ML models, prevent overfitting and underfitting, and minimize the complexity of the model. In this way, grid search techniques have been used to optimize the hyperparameters for LIGHT GBM, XGBoost, and random forests (RFs) in order to achieve improved performance, efficiency, and precision. This technique is used to determine the efficacy of every combination of the specified hyperparameters and associated value ranges, and thereafter selects the optimal hyperparameter values. Moreover, a portion of the data samples are kept entirely masked from the models and utilized only as “testing set” to improve the results of the ML models and prevent the occurrence of underfitting and overfitting. The optimized hyperparameters of our analysis indicated improved results for identifying and predicting ultimate capacity of FRP laminates bonded to concrete. The hyperparameters for RF regression, LIGHT GBM, and XGBoost are listed in Table 1, Table 2 and Table 3, respectively. 

### 2.3. Experimental Database 

The descriptive statistics of the inputs as well as the target variable employed in the investigation are depicted in Table 4. The ultimate capacity (P, kN) of FRP laminates with a concrete prism was treated as the target variable, and the input variables included the FRP’s elastic modulus x the thickness of the fiber (*E_f_T_f_*, GPa-mm), the width of the FRP laminate (*b_f_*, mm), the concrete’s compressive strength (*f_c_*′, MPa), the width of the groove (*b_g_*, mm), and the depth of the groove (*h_g_*, mm). The database contained 136 tested specimens of single-lap shear tests (SSTs) that were obtained from a previous work [40], as already reported in [24]. Between the two extremes, the data were evenly dispersed. *E_f_T_f_* had a skewness of 0.58 and a range of 12.90 to 78.90. The database used in this study was originally created by Moghaddas et al. [40]. The original experiments on FRP laminates bonded to concrete prism sheets used four different widths of FRP plates, i.e., 30, 40, 50, and 60 mm (*b_f_*, as shown in Figure 1b). The effects of four different groove sizes (i.e., 5 × 5, 5 × 10, 10 × 10, and 10 × 15) mm^2^ were also investigated. To demonstrate changes in concrete strength, three distinct mix designs with concrete strengths of 25, 35, or 45 MPa were used in accordance with ACI 211.1-91. The gradation and quality of coarse and fine aggregates were compiled as per ASTM C33/C33M. As shown in Figure 1b, the SST tests were performed on FRP plates that were coupled to a concrete prism on one side (150 × 150 × 350 mm). It is important to note that this study was based on experimental tests on FRP sheets made of the Sika wrap-200C, Sika wrap-300C, and Sika wrap-430G types of carbon and glass fibers bonded with epoxy Sikadur 330 adhesive material, as reported by Moghaddas et al. [40]. Single-lap shear tests were performed with the help of a specially formulated machine, and a hydraulic jack was utilized for the application of uniform tensile force with controlled displacements at a rate of 2 mm/min. Moreover, the tensile force exerted on the sample was accurately determined by deploying an S-type load cell.

### 2.4. Model Evaluation 

The performance of the developed models was evaluated using frequently used statistical indices, including the coefficient of determination (R^2^), root-mean-square error (RMSE), mean absolute error (MAE), relative squared error (RSE), relative root-mean-square error (RRMSE), Nash–Sutcliffe efficiency (NSE), and performance index (ρ), in accordance with previous literature [41,42,43].

## 3. Results and Discussion

### 3.1. Performance of the Developed Models

**Figure 2:**
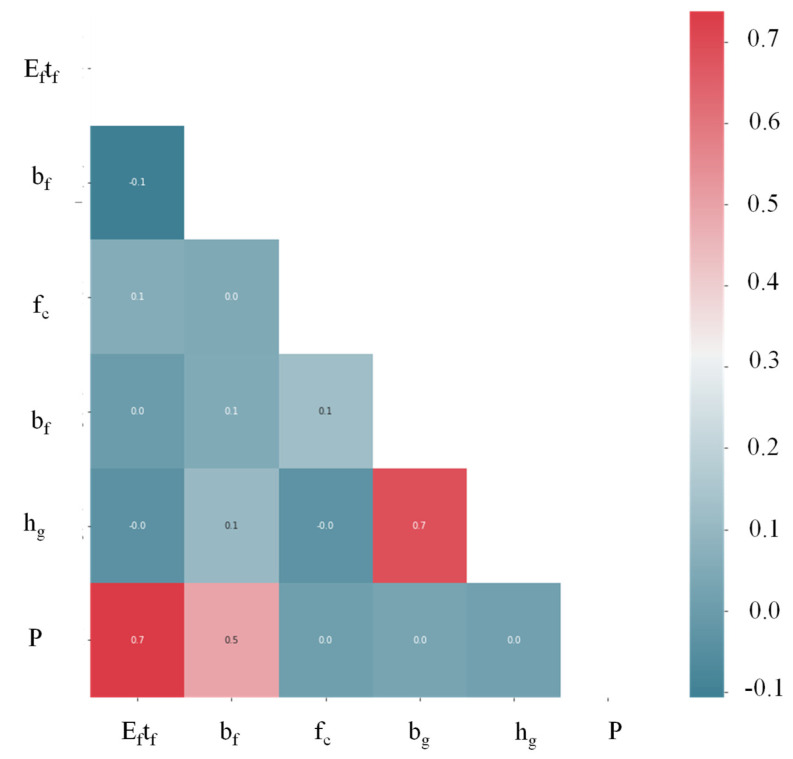
Pearson’s linear correlation for the data used in the model’s development.

Correlations between the variables in the datasets used in this study were examined using Pearson’s linear correlation. Figure 2 shows the correlation between the input and output variables. The results showed little or no linear correlation between the input and target variables, revealing the existence of a nonlinear relationship between these variables. In addition, relationships existed between *h_g_* and *b_g_*, *P* and *E_f_T_f_*, and *P* and *b_f_*. Only *b_g_* elicited a slight correlation with *f_c_*′. Using the training and validation data, we determined how well the model performed by plotting the slope of the regression line between the experimental and predicted observations. Furthermore, the predicted/experimental ratio proved to be useful for assessing the models’ performance.

#### 3.1.1. Statistical Analysis

To predict the interfacial bond strength of FRP laminates with a concrete prism and its grooves, we evaluated the robustness, effectiveness, and relative analysis of the RF, XGBoost, and LIGHT GBM models. When comparing robust performance and strongly linked models, the distribution of data points must have a slope higher than 0.8 [44], a minimal error index (MAE, NSE RSE, RMSE, and RRMSE) [45], an R^2^ greater than 0.8 [46], and a performance index close to zero [47]. Table 5 summarizes the statistical evaluation results of the three adopted ML techniques using the following performance and error metrics: R^2^, RMSE, MAE, RAE, NSE, and ρ. Using the R^2^, the minimal values for the training and testing data—0.899 and 0.820, respectively—were recorded with XGBoost and RF regression, respectively, with LIGHT GBM recording the maximal values for both the training and testing datasets. For the other metrics measuring errors in the predicted values, the minimal values were shared between the RF regression and LIGHT GBM models. The RF regression recorded minimal errors in terms of RMSE, RRMSE, and NSE, while MAE and RSE were minimal with LIGHT GBM, for both the training and testing datasets. The performance index (ρ) revealed that LIGHT GBM and RF regression had the best performance in the training and testing phases, respectively. The results of the statistical analysis revealed a close agreement between the experimental and predicted values amongst the three models. However, LIGHT GBM performed the best overall, with the highest recorded R^2^ and error parameters very close to zero, making it a reliable predictor of interfacial bond strength between the FRP laminates, in close agreement with previous findings [44].

**(i)**  ** *Comparison of regression slopes* **

**Figure 3:**
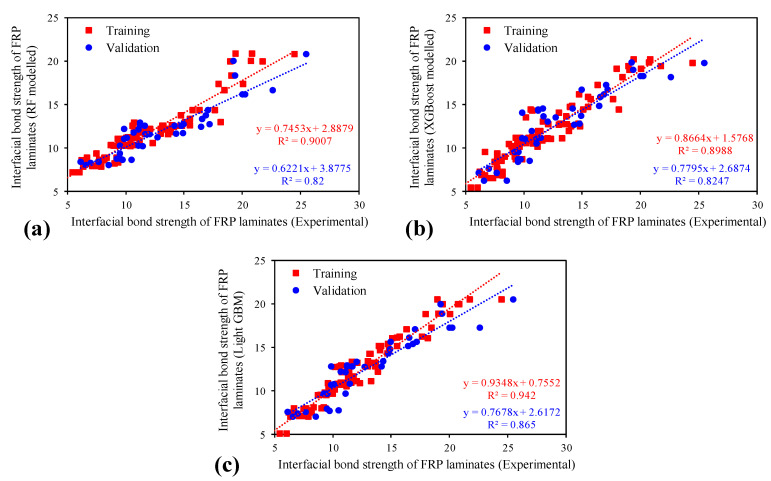
Comparison of the regression slopes’ trends between experimental and predicted values for the developed models: (**a**) RF, (**b**) XGBoost, and (**c**) LIGHT GBM.

Figure 3 reveals the cross-plots between the predicted results of the three proposed models (RF, XGBoost, and LIGHT GBM) and the experimental observations. By closely observing the slope of the regression line for the training dataset, the intensity of correlation between the results of the proposed models and the experimental data increased from XGBoost (0.8988) to RF regression (0.9007), and then to LIGHT GBM, which was the closest to the slope of an ideal regression line (1:1). As for the validation dataset, the trend was similar to that observed for the training dataset, except that there was a similar correlation between the RF regression and XGBoost models, with LIGHT GBM giving the best fit, with a regression line slope of 0.865 as compared to 0.82 (RF) and 0.8247 (XGBoost).

**(ii)**  ** *Error analysis* **

**Figure 4:**
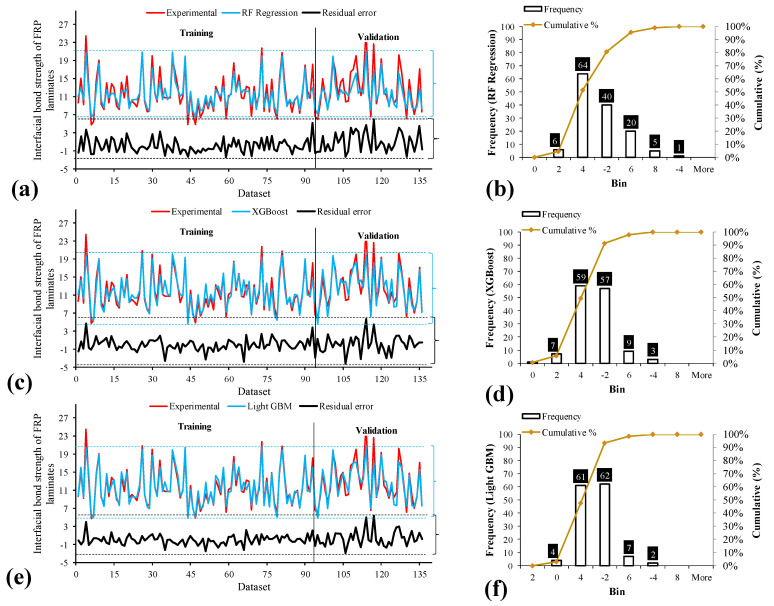
Tracing of experimental results by the prediction: (**a**) RF, (**c**) XGBoost, (**e**) LIGHT GBM and experimental to predicted ratio of: (**b**) RF, (**d**) XGBoost, (**f**) LIGHT GBM.

We also analyzed the errors from the model predictions, and the results were plotted for the training and testing datasets, as shown in Figure 4a,c,e. These plots allow us to see the residual errors between the predicted and actual experimentally observed values, in addition to the range of these error values. In addition, the histogram with frequency shows the value counts and the accompanied bin width (i.e., range of errors) for each of the proposed models. The histogram (Figure 4b,d,f) reveals that the model with the most of its dataset within the boundaries of the least errors is the LIGHT GBM, as shown in Figure 4f compared with Figure 4b,d for RF regression and XGBoost, respectively. This suggests that most of the errors between the predicted and observed values are concentrated or scattered near the zero region for all three models; however, the errors closest to zero were recorded with the LIGHT GBM.

**(iii)**  ** *Predicted-to-experimental ratio analysis* **

**Figure 5:**
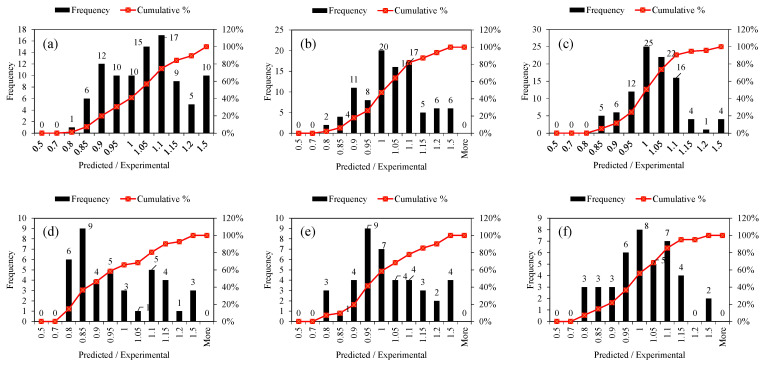
Frequency histograms of predicted-to-experimental ratios for the: (**a**,**d**) RF regression, (**b**,**e**) XGBoost, and (**c**,**f**) LIGHT GBM models for training and validation, respectively.

As part of the statistical methods used to evaluate the models’ performance, the ratio of the predicted values to the experimental values was also used so as to clearly highlight the accuracy of the model in more detail. Some researchers [1] predicted the shear strength of squat-reinforced concrete walls within ±20% of the predicted/experimental ratio when they used the XGBoost model. In conjunction with other statistical evaluations, this model resulted in a higher interpretation of accuracy than that of other empirical models. In this study, Figure 5 and Table 6 show the percentage of errors in the prediction of each of the proposed models (RF, XGBoost, and LIGHT GBM). As for the RF model, Table 6 and Figure 5 show that for the training and testing datasets, [67%, 83%, and 89%] and [43%, 76%, and 93%] of the datasets lie in predicted-to-experimental ratio ranges of [0.90–1.10], [0.85–1.15], and [0.80–1.20], respectively. In the same ranges of the predicted-to-experimental ratio, for XGBoost, [76%, 85%, and 94%] and [68%, 78%, and 90%] of the training and testing datasets were in the [0.90–1.10], [0.85–1.15], and [0.80–1.20] ratio ranges, respectively. The highest percentages of the datasets lying in the predicted-to-experimental ratio range were found with the LIGHT GBM model for both the training and testing datasets.

**(iv)**  ** *Taylor Diagrams* **

**Figure 6:**
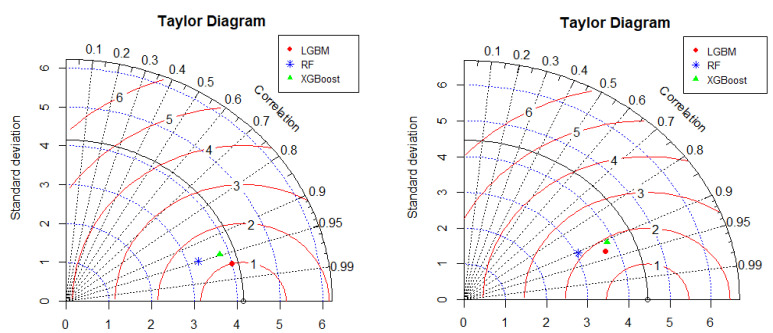
Comparison of the developed models using Taylor diagrams (LHS: training, RHS: testing).

In Figure 6, the dashed radial lines (blue) denote the standard deviation (SD), the dashed straight lines (black) denote the correlation coefficient (CC), and the continuous radial lines (red) indicate the centered root-mean-square deviation (CRMSD) between the training and testing datasets and the experimental dataset. The Taylor diagrams (shown in Figure 6) for the training and testing datasets provide more visualization of the accuracy of all of the proposed models using correlations and errors between the predicted and experimental values. These diagrams statistically summarize the data to assess the degree to which the observed and estimated values correspond based on root-mean-square error, standard deviation, and Pearson’s correlation coefficient [2]. The Taylor diagrams provide a visual summary of the predictive abilities of the proposed models in one image. They illustrate how close the experimental and predicted results are in terms of their correlation and biasness ratio [3]. The reference model is indicated by the white circular dot, with a measured SD of 4.2, CC of unity, and CRMSD of zero. For the LIGHT GBM, RF, and XGBoost models, the CC, CRMSD, and SD are approximately [0.97, 1, and 4], [0.95, 1.5, and 3.2], and [0.952, 1.3, and 3.8], respectively, for the training dataset. As for the testing phase, the CC, CRMSD, and SD are [0.93, 1.8, and 3.6], [0.90, 2.1, and 3.1], and [0.91, 1.9, and 3.7] for LIGHT GBM, RF, and XGBoost, respectively. Consequently, based on the statistical indices and external validation criteria, it can be stated with sufficient confidence that among all of the applied ML models, the LIGHT GBM model achieves the highest accuracy in predicting the interfacial bond strength of FRP laminates bonded with concrete prisms on grooves.

#### 3.1.2. SHAPASH Analysis

**Figure 7:**
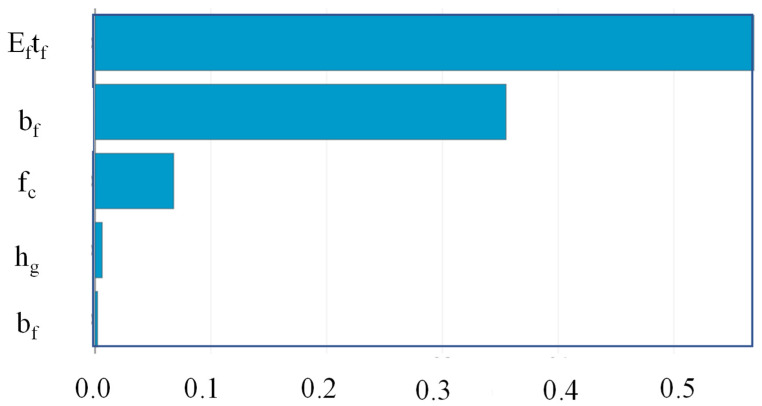
Feature importance of input variables using the SHAPASH library.

**Figure 8:**
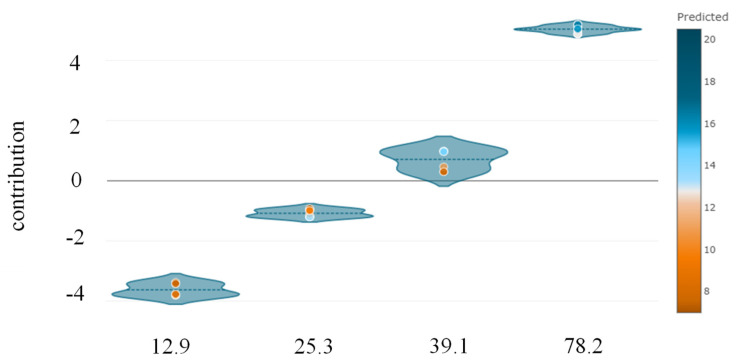
Feature contribution of *E_f_T_f_* towards the target variable.

**Figure 9:**
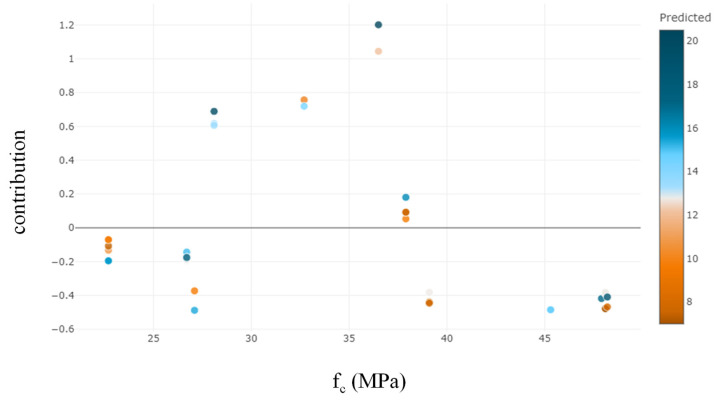
Feature contribution of *f_c_*′ towards the target variable.

**Figure 10:**
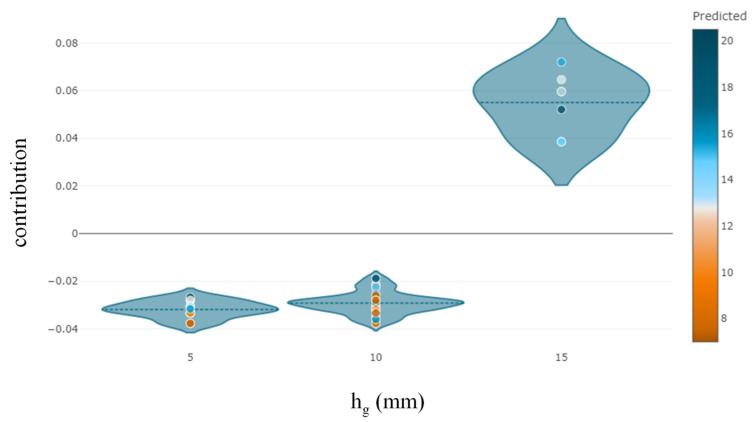
Feature contribution of *h_g_* towards the target variable.

**Figure 11:**
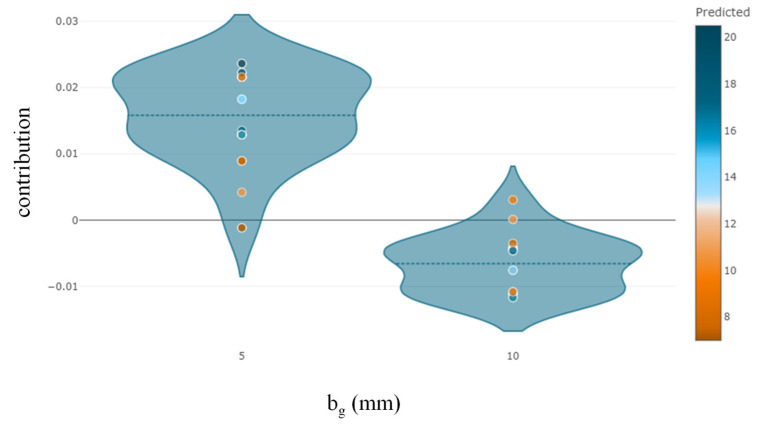
Feature contribution of *b_g_* towards the target variable.

The Python “SHAPASH” package was used to determine the relative relevance, direction of influence, and nature of influence of predictors on the target variable. It can be observed that *E_f_T_f_* is the most significant variable, followed by *bf*, *f_c_*′, *hg*, and *bg* (Figure 7). The observations of feature importance reveal concurrence with the results obtained in a previous study [2]. It is evident from Figure 8 that increases in the value of *E_f_T_f_* positively contribute to the prediction. The lowest prediction of the ultimate IBS capacity was observed at 8 kN for the value of *E_f_T_f_* at 12.9 GPa-mm, whereas the highest prediction was obtained at 78.2 GPa-mm. Similarly, the highest prediction of IBS was obtained at high compressive strength (Figure 9). Increasing the depth of the groove beyond 15 mm predicted IBS in the range of 13–20 kN (Figure 10). The specimens with a narrower groove yielded better IBS results compared to those with a wider groove (Figure 11). 

## 4. Conclusions

FRP laminates are widely utilized to retrofit a variety of reinforced concrete elements (i.e., beams, columns, joints, and slabs); therefore, it is crucial to assess their bond strength with concrete structural members. Single-lap shear strength tests, performed on the FRP laminates bonded to a concrete prism and its grooves, were used to develop three ensemble models—namely, random forest (RF) regression, extreme gradient boosting (XGBoost), and Light Gradient Boosting Machine (LIGHT GBM) models—based on machine learning (ML). It is notable that the developed models were applicable for the extreme values of the input variables used in the present study. It is also worth mentioning that the tested specimens included single-lap-sheared samples bonded to the concrete using Sikadur 300 epoxy as an adhesive material. The following conclusions can be drawn from this research:

- While investigating the optimization of the formulated models, the learning rate (0.1), maximal depth (7), and number of trees (90) were found to govern the final RF predictions. The same magnitude of optimal learning rate was obtained for the other two ML methods (i.e., XGBoost and LIGHT GBM) as well. In contrast to the RF regression, the maximal tree depth was found to be 3 for the other two models.
- The sensitivity via SHAPASH analysis indicated that *E_f_T_f_* is the most prominent input attribute, followed by the width of the FRP laminates. This is in good agreement with the Pearson’s linear correlation yielded for these two parameters as well as the ultimate axial capacity of FRP laminates. This suggests validation of the formulated models and consistency in terms of the importance of the variables using different statistical evaluation methods.
- Moreover, all of the models showed reliable performance in terms of correlation and error evaluation; however, LIGHT GBM outclassed the other two models. In LIGHT GBM, the values of R, RMSE, and MAE were 0.942, 3.40, and 0.80 for the training data, respectively, and 0.865, 3.56, and 1.3 for the testing data, respectively. For the training and validation datasets, the slopes of the regression lines were 0.9348 and 0.7678, respectively. This shows that the experimental and predicted values were in close agreement with one another.

The LIGHT GBM framework exhibits robust training speed, greater efficacy, higher accuracy, and is capable of handling large-scale data. It is also highly applicable in the binary and multi-classification problems. However, the associated drawbacks include overfitting and compatibility with the datasets. In addition, the SHAPASH analysis is highly versatile (used for plotting interactions between the considered variables alongside a training model that can be used for future predictions) and works with the aforementioned classification problems as well as with regression problems. 

## Figures and Tables

**Table 1:** Tuned or optimized hyperparameters of RF regression.

Parameters	Description	Value	Range
Learning rate	To reduce the gradient step	0.1	0.001–0.1
Maximum depth	Depth of tree	7	2–7
Number of trees	Constructing the maximum number of trees possible	90	30–150

**Table 2:** Tuned or optimized hyperparameters of XGBoost.

Parameters	Description	Value	Range
Learning rate	To reduce the gradient step	0.1	0.01–0.3
Maximum depth	Depth of tree	3	1–8
Number of estimators	Constructing the maximum number of trees possible	600	100–800
Minimum child weight	Minimum sum of instance weight (Hessian) needed in a child	3	1–5
Colsample_bytree	Subsample ratio of columns when constructing each tree	0.50	0.1–0.6

**Table 3:** Tuned or optimized hyperparameters of LIGHT GBM.

Parameters	Description	Value	Range
Learning rate	To reduce the gradient step	0.1	0.01–0.3
Maximum depth	Depth of tree	3	1–6
Number of estimators	Constructing the maximum number of trees possible	500	100–800
Colsample_bytree	Subsample ratio of columns when constructing each tree	0.60	0.1–1
Number of Leaves	Maximum number of leaves	6	1–8

**Table 4:** Descriptive statistics of the collected dataset.

Descriptive Statistic	Inputs					Target Variable
	Elastic Modulus of FRP × Thickness of FRP, *E_f_T_f_*	Width of FRP, *b_f_*	Concrete Compressive Strength, *f_c_*′	Width of Groove, *b_g_*	Depth of Groove, *h_g_*	Ultimate Capacity, *P*
Unit	GPa × mm	mm	MPa	mm	mm	kN
Range	65.30	30.00	25.50	5.00	10.00	20.73
Minimum	12.90	30.00	22.70	5.00	5.00	4.76
Maximum	78.20	60.00	48.20	10.00	15.00	25.49
Mean	40.33	46.10	33.72	7.94	10.33	12.05
Standard deviation	25.41	11.81	8.49	2.47	3.45	4.32
Sample variance	645.42	139.52	72.15	6.10	11.93	18.65
Kurtosis	−1.23	−1.49	−1.11	−1.90	−0.88	0.30
Skewness	0.58	−0.13	0.49	−0.36	−0.09	0.80
Count	136.00	136.00	136.00	136.00	136.00	136.00
Confidence level (95.0%)	4.31	2.00	1.44	0.42	0.59	0.73

**Table 5:** Performance index values of the LIGHT GBM and XGBoost models.

	Performance Index	RF Regression	XGBoost	LIGHT GBM
Training	R^2^	0.900	0.899	0.942
	RMSE	3.39	3.41	3.40
	MAE	1.2	1.0	0.8
	RSE	0.1263	0.1024	0.0580
	RRMSE	0.2931	0.2942	0.2938
	NSE	0.8737	0.8976	0.9420
	ρ	0.1504	0.1510	0.1491
Testing	R^2^	0.820	0.825	0.865
	RMSE	3.47	3.60	3.56
	MAE	1.9	1.4	1.3
	RSE	0.2871	0.1799	0.1554
	RRMSE	0.2643	0.2737	0.2713
	NSE	0.7130	0.8201	0.8446
	ρ	0.1387	0.1434	0.1406

**Table 6:** Frequencies of predicted-to-experimental ratios for the developed models.

Predicted-to-Experimental Ratio		RF		XGBoost		LIGHT GBM	
		Training	Validation	Training	Validation	Training	Validation
0.90–1.10	Frequency	64	18	81	29	72	28
	Percentage	67.37	43.90	75.79	68.29	85.26	70.73
0.85–1.15	Frequency	79	31	90	36	81	32
	Percentage	83.16	75.61	85.26	78.05	94.74	87.80
0.80–1.20	Frequency	85	38	91	39	89	37
	Percentage	89.47	92.68	93.68	90.24	95.79	95.12

## Data Availability

The data used in this research have been properly cited and reported in the main text.
